# The Relationship Between Cerebrovascular Injuries and Craniomaxillofacial Fractures: Findings From a Tertiary Hospital in Saudi Arabia

**DOI:** 10.7759/cureus.17959

**Published:** 2021-09-14

**Authors:** Ibrahim Al Babtain, Mohammed Abdullah M Alsahly, Ahmed Bandar A Aba Alkhail, Jehad T Almutib, Rakan Ahmed F Al Otaibi, Abdullah Saad H Alsalamah, Yahya M Asseri, Ahmed O Ahmed

**Affiliations:** 1 Department of General Surgery, King Abdulaziz Medical City, Riyadh, SAU; 2 Medicine, King Saud Bin Abdulaziz University for Health Sciences, Riyadh, SAU; 3 Medicine, College of Medicine, King Saud Bin Abdulaziz University for Health Sciences, Riyadh, SAU; 4 Surgery, College of Medicine, King Saud Bin Abdulaziz University for Health Sciences, Riyadh, SAU; 5 Radiology, King Abdulaziz Medical City, Riyadh, SAU

**Keywords:** blunt cerebrovascular injury, craniomaxillofacial fracture, skull fractures, facial fractures, internal carotid artery, vertebral artery

## Abstract

Background and objective

Blunt cerebrovascular injuries (BCVIs) can lead to serious outcomes, particularly because they are difficult to detect in the acute phase. There are studies that have described the association between cerebrovascular injuries and craniomaxillofacial (CMF) fractures; however, no such study has been conducted among a Saudi population. In light of this, we conducted this study to evaluate the correlation between BCVI and CMF fractures among the local population in Saudi Arabia. In addition, the most common types of fractures associated with BCVI were identified.

Methods

This retrospective cohort study was conducted at the King Abdulaziz Medical City, a tertiary hospital in Riyadh, Saudi Arabia. All eligible patients with CMF fractures who were hospitalized at the King Abdulaziz Medical City were included. Consecutive patients were screened; no sampling or randomization was required. Patients with penetrating or avulsive mechanisms of injury were excluded.

Results

Out of a total of 1,560 patients included in the study, 1,537 (98.5%) had CMF fractures, while 23 (1.5%) had BCVIs. None of the patients with CMF fractures had BCVIs. Among the patients with BCVIs, 12 (52.2%) were men and 11 (47.8%) were women. The mean age of these patients was 46.91 ± 17.04 years. Among patients with CMF fractures, 1,071 (69.7%) were men and 466 (30.3%) were women. Their mean age was 23.93 ± 17.36 years.

Conclusion

The study did not identify any correlation between BCVI and CMF fractures; however, further studies with larger samples across multiple centers are needed to validate our findings and gain deeper insight into the relationship between BCVI and CMF fractures.

## Introduction

Blunt trauma can cause vascular injury to the vertebral and internal carotid arteries. Blunt cerebrovascular injuries (BCVIs) secondary to craniomaxillofacial (CMF) fractures are usually difficult to detect in an acute setting and hence may lead to a delay in diagnosis [1-3]. Although BCVI is considered rare, accounting for only 1% of all blunt trauma cases, it has an estimated stroke-related mortality rate of 21% [3-6]. Early intervention plays a major role in reducing cerebrovascular insult, from 20% to 9.6% as per some studies [2,4,7]. Despite the devastating consequences of BCVI, controversy still exists with respect to identifying the population at risk of this injury [2,8-9]. The association between BCVI and CMF fractures has been demonstrated in several previous studies [2,10-12]. Studies about the patterns of CMF fractures have shown varying results due to age as well as social and ethnic diversity among the study populations [13-15]. Hence, we believe there is a need to continually update the fracture patterns of injury. The aim of this study was to update the information on fracture patterns in the capital region of Riyadh, Saudi Arabia, and to determine whether there is an association between CMF fractures and BCVI.

## Materials and methods

Study design and settings

This was a retrospective cohort study conducted to assess the relationship between BCVI and CMF fractures among hospitalized trauma patients at the King Abdulaziz Medical City, Ministry of National Guard Health Affairs, Riyadh, the Kingdom of Saudi Arabia. With a bed capacity of 1,501, the King Abdulaziz Medical City is one of the largest tertiary care centers in the Kingdom. It serves the National Guard personnel and their families.

Study population

The study included all consecutive patients with CMF fractures and patients with BCVI who were admitted to the King Abdulaziz Medical City between January 2016 and December 2020. Patients with penetrative injuries and avulsion as the mechanism of injury as well as those without a CT scan for review were excluded.

Data collection

Ethical approval and data were provided by the King Abdullah International Medical Research Center. Data were extracted from the BESTCare system, the digital hospital information system used at the King Abdulaziz Medical City, and then entered into and coded using Microsoft Excel. The collected data included patient demographics such as age, sex, type of fracture, and type of BCVI, which was determined by two radiologists after reviewing patients’ CT scans. BCVI was classified into five grades based on the Denver scale. The type of fracture data provided was as follows: upper facial (frontal bone, frontal sinus, orbital roof), midfacial (Le Fort I-III, naso-orbito-ethmoidal, zygomatic, orbital other than the roof, maxillary, nasal), lower facial, basilar (sphenoid, temporal, petrous, occipital, unspecified), combined facial fracture, combined skull fracture, and other fractures.

Statistical analysis

Statistical analysis was performed using SPSS Statistics version 23 (IBM Corp., Armonk, NY). Categorical variables are presented as frequencies and percentages. Numerical variables are calculated as means and standard deviations. The Chi-square test was used to test for the presence of an association between categorical variables. The independent t-test was also used to test for the association. A p-value less than 0.05 was considered statistically significant.

## Results

**Figure 1:**
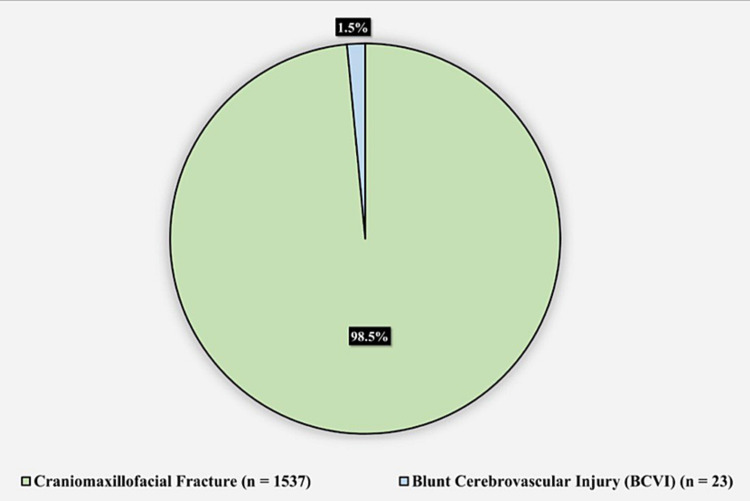
Diagnoses of the patients (n=1,560)

A total of 1,560 patients were included in the study. Figure 1 shows the diagnosis summary of the patients; 1,537 (98.5%) patients had CMF fractures, while 23 (1.5%) had a BCVI. None of the patients with CMF fractures had a BCVI.

Table 1 presents the sociodemographic characteristics of the included patients. Among the patients with BCVI, 12 (52.2%) were men and 11 (47.8%) were women, with an overall mean age of 46.91 ± 17.04 years. Among the patients with CMF fractures, 1,071 (69.7%) were men and 466 (30.3%) were women, with an overall mean age of 23.94 ± 17.40 years.

**Table 1:** Sociodemographic characteristics of patients (n=1,560)

Patient characteristics		
Blunt cerebrovascular injury (BCVI) (n=23)	N	%
Sex		
Male	12	52.2
Female	11	47.8
Age (years)		
Mean	46.91	
Standard deviation	17.04	
Craniomaxillofacial fracture (n=1,537)	N	%
Sex		
Male	1,071	69.7
Female	466	30.3
Age (years)		
Mean	23.94	
Standard deviation	17.4	

Table 2 displays the CMF fracture profiles. Among the 31 patients with upper third facial fractures, nine (0.59%) had frontal bone fractures, seven (0.45%) had frontal sinus fractures, and 15 (0.98%) had orbital roof fractures. Among the 777 patients with midfacial fractures, seven (0.45%) had Le Fort I fractures, three (0.2%) had Le Fort II fractures, two (0.13%) had Le Fort III fractures, 19 (1.24%) had naso-orbito-ethmoidal fractures, 68 (4.42%) had midfacial zygomatic fractures, 62 (4%) had an orbital other than the roof fracture, 47 (3.06%) had a maxillary fracture, and 595 (38.71%) had a nasal fracture. As for the basilar skull fracture types, one (0.07%) patient had a sphenoid fracture, 36 (2.3%) had temporal fractures, 10 (0.7%) had basilar occipital fractures, and 19 (1.2%) had unspecified skull fractures. Of all the patients, 273 (17.8%) had unspecified facial and skull fractures.

**Table 2:** Summary of the various types of craniomaxillofacial fracture

Location of fracture		N	%
Upper facial fracture (n=31)			
Frontal bone		9	0.59
Frontal sinus		7	0.45
Orbital roof		15	0.98
Midfacial fractures (n=803)			
Le Fort I		7	0.45
Le Fort II		3	0.2
Le Fort III		2	0.13
Naso-orbito-ethmoidal		19	1.24
Zygomatic		68	4.42
Orbital other than the roof		62	4
Maxillary		47	3.06
Nasal		595	38.71
Basilar skull fractures (n=61; however, the total adds up to 66)			
Sphenoid		1	0.07
Temporal		36	2.3
Basilar of occipital		10	0.7
Unspecified		19	1.2
Facial and skull unspecified		273	17.8
Other (n=136)			
Teeth		136	8.8

**Figure 2:**
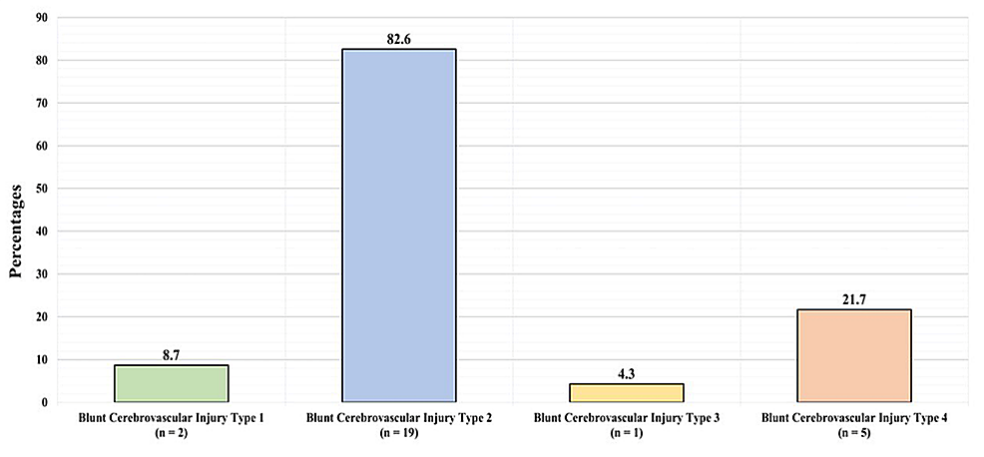
Blunt cerebrovascular injury types

Figure 2 shows the types of BCVI among these patients. Two patients (8.7%) had type 1 BCVI, 19 (82.6%) had type 2, one (4.3%) had type 3, and five (21.7%) patients had type 4.

**Figure 3:**
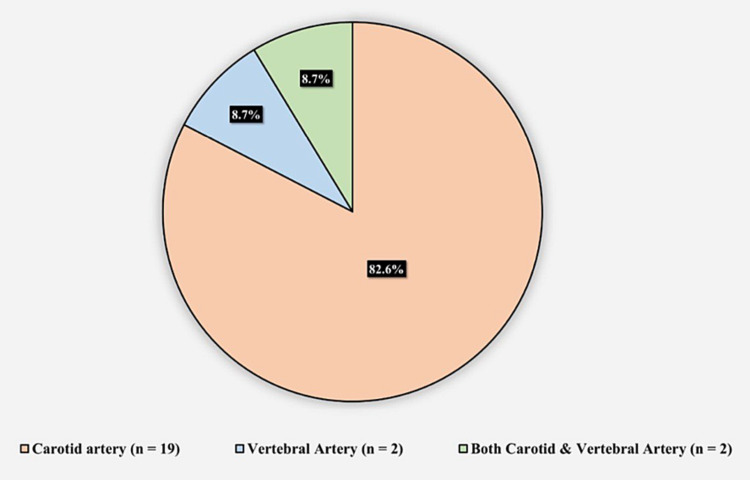
Affected arteries in patients with blunt cerebrovascular injuries

Figure 3 presents the affected arteries among patients with BCVI. The carotid artery was affected in 19 (82.6%) patients, the vertebral artery was affected in two (8.7%), and both the carotid and vertebral artery were affected in two (8.7%) patients.

**Figure 4:**
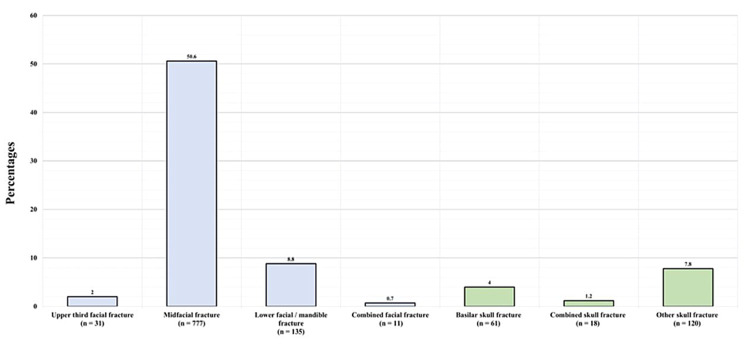
Craniomaxillofacial fracture types

Figure 4 illustrates the fracture types in patients with CMF fractures. Thirty-one patients (2%) had upper third facial fractures, 777 (50.6%) had midfacial fractures, 135 (8.8%) had lower face/mandible fractures, 10 (0.7%) had combined facial fractures, 61 (4%) had basilar skull fractures, 18 (1.2%) had combined skull fractures, and 120 (7.8%) had other skull fractures. Among the patients with CMF fractures, 176 (11.4%) also had tooth fractures.

**Figure 5:**
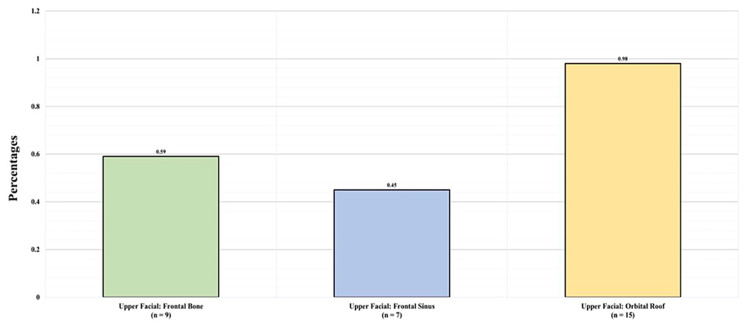
Upper facial fracture types

Figure 5 shows the types of upper facial fractures detected in the study. Among the 31 patients with upper third facial fractures, nine (0.59%) had frontal bone fractures, seven (0.45%) had frontal sinus fractures, and 15 (0.98%) had orbital roof fractures.

**Figure 6:**
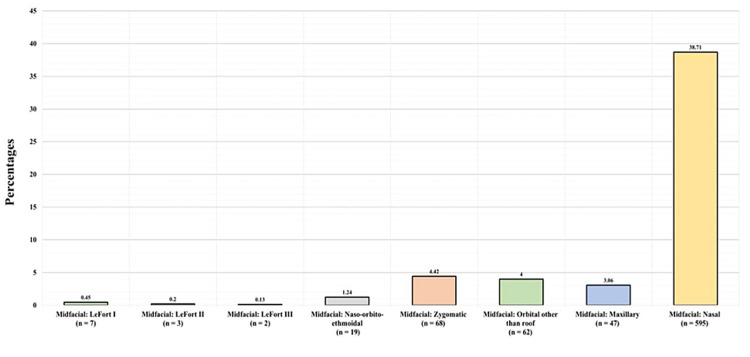
Midfacial fractures

Figure 6 presents the midfacial fracture types detected in the study participants. Among the 777 patients with midfacial fractures, seven (0.45%) had a Le Fort I fracture, three (0.2%) had a Le Fort II fracture, two (0.13%) had a Le Fort III fracture, 19 (1.24%) had a naso-orbito-ethmoidal fracture, 68 (4.42%) had a midfacial zygomatic fracture, 62 (4%) had orbital other than the roof fracture, 47 (3.06%) had a maxillary fracture, and 595 (38.71%) had a nasal fracture.

**Figure 7:**
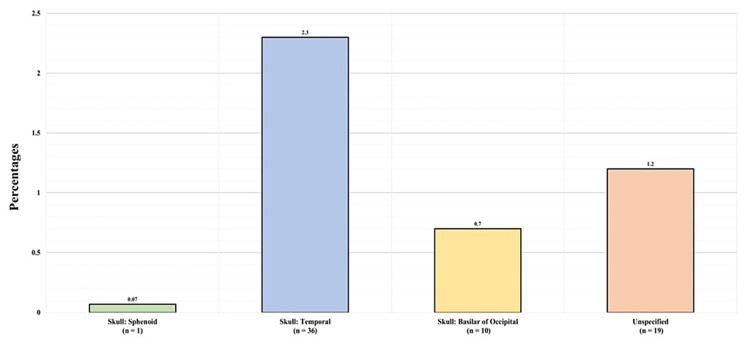
Basilar skull fractures

Figure 7 shows the basilar skull fracture types. One (0.07%) patient had a sphenoid fracture, 36 (2.3%) had a temporal fracture, 10 (0.7%) had a basilar occipital fracture, and 19 (1.2%) had an unspecified skull fracture.

Table 3 shows the factors associated with BCVI. Age was significantly associated with BCVI (p<0.001). Patients with BCVI had significantly higher mean age than those without BCVI (46.91 ± 17.04 vs. 23.94 ± 17.40 years, respectively). Sex was not significantly associated with BCVI.

**Table 3:** Factors associated with blunt cerebrovascular injury *Significant at level <0.05 SD: standard deviation

Factor	Blunt cerebrovascular injury		P-value
	No	Yes	
Sex, n (%)			0.07
Male	1,071 (98.9%)	12 (1.1%)	
Female	466 (97.7%)	11 (2.3%)	
Age (years), mean + SD	23.94 + 17.40	46.91 + 17.04	<0.001*

## Discussion

The findings of epidemiological studies are largely dependent on the demographics of the study population, including socioeconomic status, geographic location, and period of the study [16]. Nevertheless, the results of our research align with those of many local and international studies. The mean age of the participants with CMF fractures in our study was 23.94 ± 17.40 years, which is similar to the average of 25.75 ± 15.5 years found in another study [17]. Upper facial fractures were the least prevalent type of CMF fractures, a finding corroborated by two other studies to date [18,19].

Furthermore, midfacial fractures were found to be the most common type of CMF fractures, accounting for more than 50.6% of all CMF fractures. This finding is supported by an Austrian study, which found that midfacial fractures were the most common facial bone fractures, accounting for more than 71.5% of their sample of 7,061 patients with bone fractures [14]. Among midfacial fractures, nasal bone fractures were the most common type, followed by zygomatic, orbital (excluding the roof), and maxillary fractures, which are comparable to findings of a 2006 study in which zygomatic fractures and nasal fractures were the most common subtypes, accounting for 24% and 23% of all midfacial fractures, respectively [15,20]. However, another study found zygoma to be the most common midfacial fracture, followed by the maxilla and nasal fractures [21]. Furthermore, a retrospective study of 2,969 patients from 12 trauma centers in Ontario, Canada, revealed that the most commonly occurring fractures were found in the maxilla and orbital regions, followed by the zygoma and nasal bone [22]; thus, although midfacial fractures are the most common type, there is variation between various midfacial fracture subtypes.

Regarding the Le Fort classification of midfacial fractures, in our study, Le Fort I was the most common type, followed by Le Fort II and III, respectively, which is similar to the results of another study that found the most common fracture type to be Le Fort I followed by Le Fort II [23]. In our study, basilar fractures accounted for 4% of all CMF fractures and 30% of all skull fractures. The temporal bone fracture was the most common basilar fracture, accounting for 55% of these types of fractures, followed by the basilar part of the occipital bone (15%) and the sphenoid bone (2%). These results are similar to those of a study that found prevalence rates of 47%, 45%, and 37% for these three fractures, respectively [24].

As the practice of trauma surgery has continued to evolve, awareness of BCVI among clinicians has increased. In this study, we investigated the incidence rate of BCVI among patients with CMF fractures in our center and found 23 incidents of BCVI, none of whom had CMF fractures. No local data regarding the relationship between BCVI and CMF fractures is available in the literature. However, several studies support our findings [2,25,26], while some others do not. A systematic review identified 410 BCVI cases among 90,968 blunt trauma admissions with CMF fractures, constituting an overall incidence of 0.45% [18]. The mean age of BCVI patients in our study was 46.91 ± 17.04 years, similar to that in Weber et al.’s study (46 ± 19 years) [25]. In our study, 52.2% of BCVI patients were male, whereas the proportion of males with BCVI reported in other studies ranged from 57.8-65.2% [2,9,27,28].

Grade 1 was the most frequently reported injury grade in a study conducted by Malhotra et al. [29]. According to Stein et al., both grades 1 and 2 were reported in similar numbers and comprised the most common BCVI grades, followed by grades 4, 3, and 5, respectively [2]. However, the most commonly reported BCVI grade in our study was grade 2, followed by grades 4, 3, 1, and 5.

There is inconsistency in the literature regarding the injured vessels in the BCVI. Berne et al. and Savoie et al. have reported more vertebral artery injuries than carotid artery injuries [9,27]. Nevertheless, the findings of our study align with those of Weber et al. and Jones et al., who found a preponderance of carotid artery injuries [25,28].

## Conclusions

Our study examined the prevalence of CMF fractures in a tertiary hospital in Riyadh, Saudi Arabia. We found that midfacial fractures were the most common type, especially nasal fractures, and we found no incidence of BCVI among patients with CMF fractures. We also found that the mean age of the patients with CMF fractures was 23.94 ± 17.40 years, while it was 46.91 ± 17.04 years for patients with BCVI. More local studies need to be conducted to further investigate the types of CMF fractures and the incidence of BCVI among them.
